# A randomized phase III study evaluating pegylated liposomal doxorubicin versus capecitabine as first-line therapy for metastatic breast cancer: results of the PELICAN study

**DOI:** 10.1007/s10549-016-4033-3

**Published:** 2016-10-31

**Authors:** Nadia Harbeck, Steffen Saupe, Elke Jäger, Marcus Schmidt, Rolf Kreienberg, Lothar Müller, Burkhard Joerg Otremba, Dirk Waldenmaier, Julia Dorn, Mathias Warm, Michael Scholz, Michael Untch, Maike de Wit, Jana Barinoff, Hans-Joachim Lück, Philipp Harter, Doris Augustin, Paul Harnett, Matthias W. Beckmann, Salah-Eddin Al-Batran, On behalf of the PELICAN Investigators

**Affiliations:** 1 Breast Center, Department of Obstetrics and Gynecology and CCC of LMU, University of Munich, Munich, Germany; 2 Department of Obstetrics and Gynecology, Technical University of Munich, Munich, Germany; 3 Oncology and Hematology, Krankenhaus Nordwest, Frankfurt, Germany; 4 Department of Obstetrics and Gynecology, Johannes Gutenberg University, Mainz, Germany; 5 Universitätsfrauenklinik, Ulm, Germany; 6 Onkologische Schwerpunktpraxis, Leer, Germany; 7 Onkologische Praxis Oldenburg, Oldenburg, Germany; 8 MSD Sharp & Dohme, Haar, Germany; 9 Brustzentrum, Krankenhaus Köln-Holweide, Cologne, Germany; 10 Trium Analysis Online GmbH, Munich, Germany; 11 Helios Klinikum Berlin-Buch, Berlin, Germany; 12 Vivantes Klinikum Neukoelln, Berlin, Germany; 13 Dr.-Horst-Schmidt-Kliniken Wiesbaden, Wiesbaden, Germany; 14 Kliniken Essen-Mitte, Essen, Germany; 15 Klinikum des Landkreises Deggendorf, Deggendorf, Germany; 16 Crown Princess Mary Cancer Centre Westmead, Sydney, Australia; 17 Department of Gynecology and Obstetrics, Comprehensive Cancer Center Erlangen-EMN, University Hospital Erlangen, Erlangen, Germany

**Keywords:** Pegylated liposomal doxorubicin, Capecitabine, Metastatic breast cancer, PELICAN

## Abstract

**Purpose:**

The PELICAN trial evaluates for the first time efficacy and safety of pegylated liposomal doxorubicin (PLD) versus capecitabine as first-line treatment of metastatic breast cancer (MBC).

**Methods:**

This randomized, phase III, open-label, multicenter trial enrolled first-line MBC patients who were ineligible for endocrine or trastuzumab therapy. Cumulative adjuvant anthracyclines of 360 mg/m^2^ doxorubicin or equivalent were allowed. Left ventricular ejection fraction of >50 % was required. Patients received PLD 50 mg/m^2^ every 28 days or capecitabine 1250 mg/m^2^ twice daily for 14 days every 21 days. The primary endpoint was time-to-disease progression (TTP).

**Results:**

210 patients were randomized (*n* = 105, PLD and *n* = 105, capecitabine). Adjuvant anthracyclines were given to 37 % (PLD) and 36 % (capecitabine) of patients. No significant difference was observed in TTP [HR = 1.21 (95 % confidence interval, 0.838–1.750)]. Median TTP was 6.0 months for both PLD and capecitabine. Comparing patients with or without prior anthracyclines, no significant difference in TTP was observed in the PLD arm (log-rank *P* = 0.64). For PLD versus capecitabine, respectively, overall survival (median, 23.3 months vs. 26.8 months) and time-to-treatment failure (median, 4.6 months vs. 3.7 months) were not statistically significantly different. Compared to PLD, patients on capecitabine experienced more serious adverse events (*P* = 0.015) and more cardiac events among patients who had prior anthracycline exposure (18 vs. 8 %; *P* = 0.31).

**Conclusion:**

Both PLD and capecitabine are effective first-line agents for MBC.

## Introduction

Metastatic breast cancer (MBC), though not considered curable by today’s therapies, deserves effective treatment. With appropriate chemotherapy, many patients derive its potential benefits, including symptom relief, maintenance of quality of life (QoL), and prolongation of survival. Much research effort has been devoted to identifying the most effective yet tolerable chemotherapy regimens for MBC. The use of sequential single-agent chemotherapy is generally less toxic than combination therapy and yields survival rates similar to those observed with multi-agent regimens [1–3]. In studies that have demonstrated a statistically significant survival benefit for combination therapy, tolerability must be considered and toxicity is often prohibitive in this palliative setting [4].

The choice among active chemotherapy agents for MBC hinges on efficacy and patient preference, particularly regarding potential side effects. Among the active agents, in particular in the current clinical setting of anthracycline and taxane pre-treatment in the adjuvant setting, are pegylated liposomal doxorubicin (PLD) and capecitabine. Both have demonstrated single-agent efficacy in MBC, are supported by established guidelines, and are relatively well tolerated [5–11].

Adequate evaluation of anthracyclines as first-line treatment for metastatic disease is required due to their routine use in the adjuvant setting. However, use of conventional anthracyclines in the palliative setting is hindered by both acute toxicities and the long-term risk of cardiotoxicity. The pegylated liposomal formulation of doxorubicin prolongs the plasma half-life and may enhance tumor localization of the drug while lowering toxicity to normal tissues. A phase III trial comparing PLD to conventional doxorubicin as first-line therapy for MBC showed comparable efficacy between the two agents with reduced cardiotoxicity in PLD-treated patients [6]. Median progression-free survival (PFS) times were 6.9 months for PLD versus 7.8 months for doxorubicin (hazard ratio [HR] 1.00; 95 %; confidence interval [CI] 0.82–1.22) and median overall survival (OS) times were 21 months for PLD versus 22 months for doxorubicin (HR = 0.94; 95 % CI 0.74–1.19). Clinical congestive heart failure (CHF) occurred in 2 patients treated with PLD and 12 patients treated with doxorubicin. The most frequent PLD-associated toxicity was palmar-plantar erythema (PPE; 48 % all grades, 17 % grade 3 or 4). With PLD having similar efficacy and a more favorable cardiotoxicity profile than conventional doxorubicin, comparing PLD to an effective non-anthracycline regimen in the first-line setting is warranted.

Capecitabine is an oral fluoropyrimidine that mimics infusional 5-fluorouracil (5-FU) and, in the presence of elevated intratumoral thymidine phosphorylase concentrations, generates 5-FU preferentially at the tumor site [12]. Capecitabine produced a response rate of 36 % and median time-to-progression of 3.0 months in anthracycline-pretreated patients [7]. As first-line therapy in women aged 55 years and older, capecitabine produced a response rate of 30 %, median time to progression (TTP) of 4.1 months, and median OS time of 19.6 months, parameters similar to the combination comparator arm of cyclophosphamide, methotrexate and 5-FU (CMF) [8]. Capecitabine was associated with grade 3–4 PPE in 15 % of patients and grade 3–4 diarrhea and stomatitis in 8 % of patients each.

The current phase III trial, PELICAN, is the first trial to evaluate the efficacy and safety of PLD versus capecitabine as first-line treatment of MBC.

## Patients and methods

### Study design

PELICAN is a randomized, phase III, open-label, multicenter trial. Patients were centrally randomized in a 1:1 ratio using a computer-generated randomization scheme that was balanced by permutated blocks and stratified according to age and prior anthracycline and/or taxane treatment.

The primary objective of the study is to compare between the two arms TTP, defined as the duration from first study drug administration to the first documented evidence of progression as assessed by the investigator or death from any cause. Secondary endpoints are to compare overall response rate, overall survival, time to treatment failure, QoL, and safety between the two treatment arms. An additional secondary endpoint, to assess the impacts of PLD and capecitabine on age- and comorbidity-related treatment burdens in all patients via geriatric assessment, will be reported in a separate publication.

### Patients

Eligible patients were women aged ≥18 years with metastatic disease of cytologically or histologically confirmed breast cancer whose clinical condition allowed monotherapy treatment or who expressed a desire to be treated with monotherapy. Other inclusion criteria included Eastern Cooperative Oncology Group (ECOG) performance status 0–2; sufficient life expectancy to receive chemotherapy, adequate renal, liver, and bone marrow function; and normal sodium and potassium serum levels.

Exclusion criteria included prior chemotherapy for metastatic disease (prior endocrine therapy was permitted); eligibility for hormone therapy (those having progressed on endocrine therapy were permitted); eligibility for trastuzumab; concomitant treatment for metastatic disease except bisphosphonates and including hormonal therapy, radiation, trastuzumab, or other biological; prior treatment with capecitabine; prior adjuvant anthracycline exceeding a cumulative dose of 360 mg/m^2^ doxorubicin or equivalent; anthracycline-resistant disease (defined as developing locally-recurrent or metastatic disease during, or relapse <12 months after completion of anthracycline therapy); central nervous system metastasis unless asymptomatic for ≥3 months; dyspnea on exertion; and cardiac disease of New York Heart Association (NYHA) Class II or greater, or clinical evidence of congestive heart failure or myocardial infarct within 6 months or a left ventricular ejection fraction (LVEF) <50 %.

### Treatment

Randomized patients received either PLD 50 mg/m^2^ every 28 days or capecitabine 1250 mg/m^2^ twice daily for 14 days every 21 days, i.e., the registered doses. Treatment continued until disease progression or unacceptable toxicity. Adjustments for grade 2 or 3 neutropenia or thrombocytopenia consisted of a treatment delay until absolute neutrophil count ≥1500 cells/mm^3^ and/or platelets ≥75,000 cells/mm^3^. Grade 4 neutropenia or thrombocytopenia necessitated delay until recovery and dose reduction to 75 % for capecitabine and 80 % for PLD. Adjustments for non-hematologic toxicities were toxicity- and drug-specific, consisting of capecitabine dose reductions to 75 or 50 % and PLD dose reductions to 80 or 60 %. Patients received supportive care per institutional guidelines. Use of erythropoietic factors and granulocyte colony stimulating factors was allowed; the protocol did not specify guidelines supporting or prohibiting prophylactic use. If LVEF decreased by ≥20 % absolute percentage points or if LVEF decreased by ≥10 % absolute percentage points and to <50 %, PLD was to be discontinued.

### Assessments

Baseline and end of study assessments included physical exam, routine laboratory tests (complete blood count and complete metabolic panel), appropriate imaging studies to assess measurable disease, multiple gated acquisition scan (MUGA) or echocardiogram (ECHO), adverse events, QoL, and ECOG performance status. On Day 1 of each cycle, assessments included physical exam, ECOG PS, routine laboratory tests, QoL, and adverse events. On Days 7–14 of each cycle, patients were monitored for adverse events, particularly skin toxicity, at the study center or by their family physician. Response assessment was based on Response Evaluation Criteria in Solid Tumors (RECIST) [13] and occurred every 3 months using the same imaging technique as at baseline. Response or stable disease was confirmed >4 and <12 weeks later. Cardiac assessment was conducted by MUGA or ECHO prior to each PLD course when the total cumulative anthracycline dose reached ≥450 mg/m^2^ doxorubicin or equivalent, upon clinical evidence of cardiac dysfunction (cardiomegaly on chest X-ray; basilar rales; S3 gallop; or either paroxysmal nocturnal dyspnea orthopnea, or significant dyspnea on exertion), or at any time per the treating physician. Quality of life was assessed using the European Organization for Research and Treatment of Cancer (EORTC) QoL Questionnaire (QLQ)-C30 [14], with an addendum of 4 questions addressing hand-foot syndrome and stomatitis, and the Subjective Significance Questionnaire (SSQ), which is designed to determine the significance to patients of changes in health-related QoL scores addressed by the EORTC QLQ-C30 [15]. After the end of treatment, patients were assessed at least every 6 months for progression, survival, and subsequent treatment.

### Statistics

The initial planned sample size of 346 was reduced to 210 patients. The reduction was based on a larger-than-initially expected difference in estimated average times to progression between the two regimens of 4 months for capecitabine and 6.9 months for PLD in the first-line setting, which were derived from data published during protocol development. [6, 8, 16–23] From these times, an estimated hazard ratio of 0.58 was calculated; however, a more conservative estimate of 0.65 was applied, requiring approximately 95 patients per arm and 210 patients total, assuming 10 % loss. Type I and type II errors were set at 0.05 and 0.2, respectively. Three planned interim safety analyses occurred during the study; the final analysis, which occurred November 30, 2010, is reported here.

Data for baseline characteristics and safety were summarized. Time to disease progression and overall survival were analyzed using the Kaplan–Meier method. The log-rank test was used to compare TTP between the two treatments. If the trial failed to detect a significant difference between the two treatments, the results of the superiority trial were to be summarized by means of a one-sided 97.5 % CI for the HR (PLD vs. capecitabine). The upper end of that CI provides a quantitative estimate of the minimum estimated effect of PLD relative to capecitabine. If this estimate fell below the margin for non-inferiority, PLD would be considered as being non-inferior to capecitabine. The prospectively defined margin for non-inferiority was a HR of 1.143 reflecting a clinically acceptable difference in TTP of 0.75 months under an expected median TTP of up to 6 months in the comparator. The overall survival curves were compared using a two-tailed log-rank statistic to test for homogeneity of survival functions. Chi square was used to compare overall response rates between groups. Efficacy analyses were performed on the intent-to-treat population, defined as all randomized patients. The safety analysis included all randomized patients who received at least a partial dose of study medication. Quality of life endpoints were analyzed with a one-way analysis of covariance (ANCOVA), with the baseline values included as a covariate.

### Ethics

The study was performed in accordance with the International Conference on Harmonisation guidelines for Good Clinical Practice. All patients provided written informed consent and Institutional Review Board approval was secured at each site. The study was sponsored by Merck, formerly Schering-Plough Corporation and Essex Pharma GmbH and is registered with European Union Drug Regulating Authorities Clinical Trials (EudraCT; Number 2005-003164-35) and clinicaltrials.gov (NCT00266799).

## Results

**Fig. 1:**
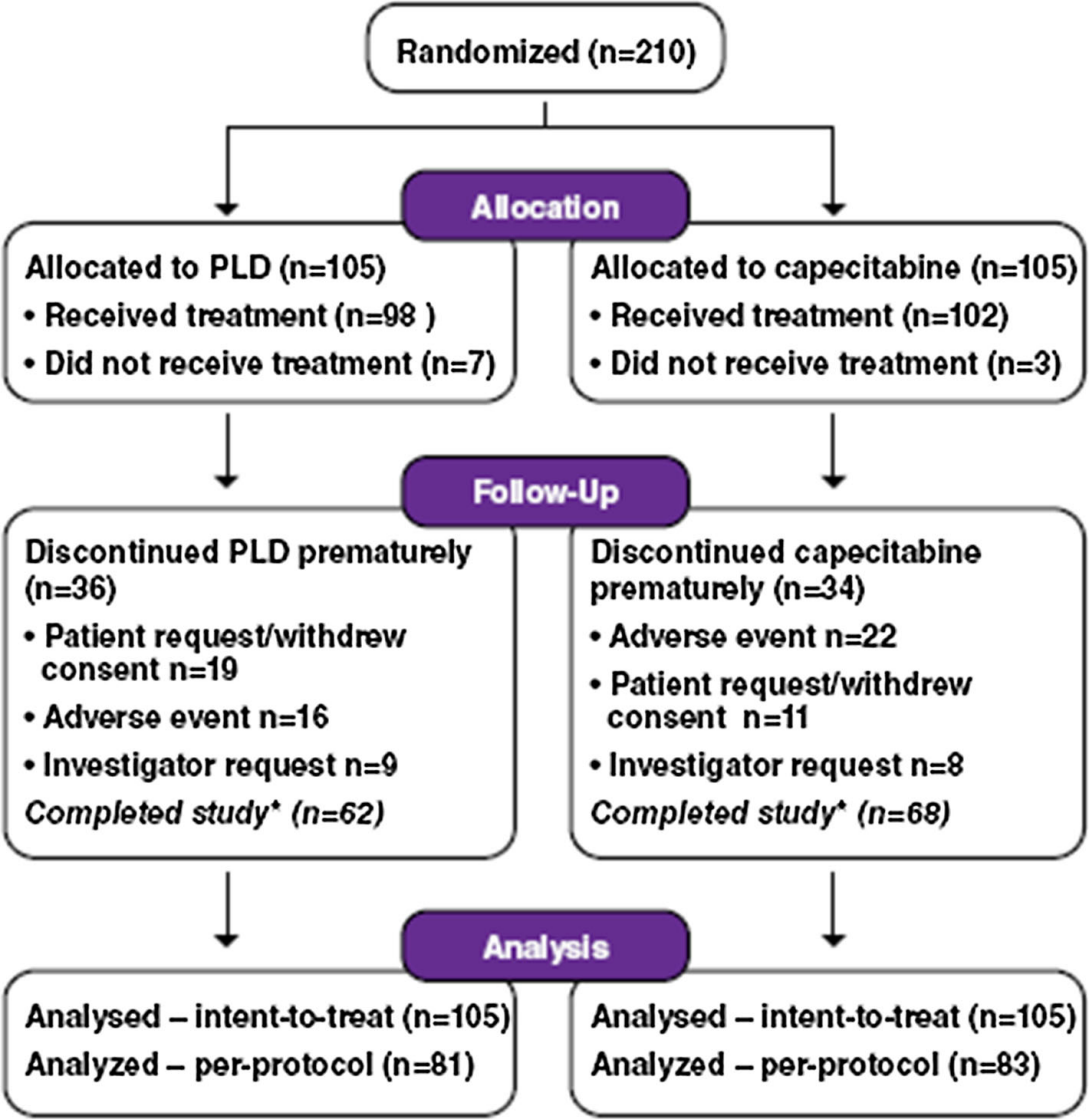
CONSORT diagram. *Completed patients were those who stopped study treatment due to progressive disease

Between January 2006 and October 2010, 210 patients were randomized (Fig. 1). The intent-to-treat analysis comprised 210 patients (*n* = 105 in the PLD arm and *n* = 105 in the capecitabine arm). Baseline characteristics were well balanced between groups (Table 1). Anthracyclines had been previously administered to 37 % of patients in the PLD arm and 36 % of patients in the capecitabine arm. Patients in both arms received a median of 5 cycles with a range of 0–24 cycles in the PLD arm and 0–41 cycles in the capecitabine arm (*P* = 0.078).

**Table 1:** Baseline characteristics

	PLD *N* = 105	Capecitabine *N* = 105
	*N* (%)	*N* (%)
Median age of patient, years (range)	62 (36–82)	63 (22–85)
ECOG performance status		
Missing	5 (4)	1 (1)
0	51 (49)	53 (50)
1	43 (41)	45 (43)
2	6 (6)	5 (5)
3	0	1 (1)
Prior anthracyclines		
Yes	39 (37)	38 (36)
No	66 (63)	67 (64)
Menopausal status		
Premenopausal	5 (5)	5 (5)
Postmenopausal	85 (83)	87 (85)
Not known/examined	15 (14)	13 (12)

### Efficacy

**Fig. 2:**
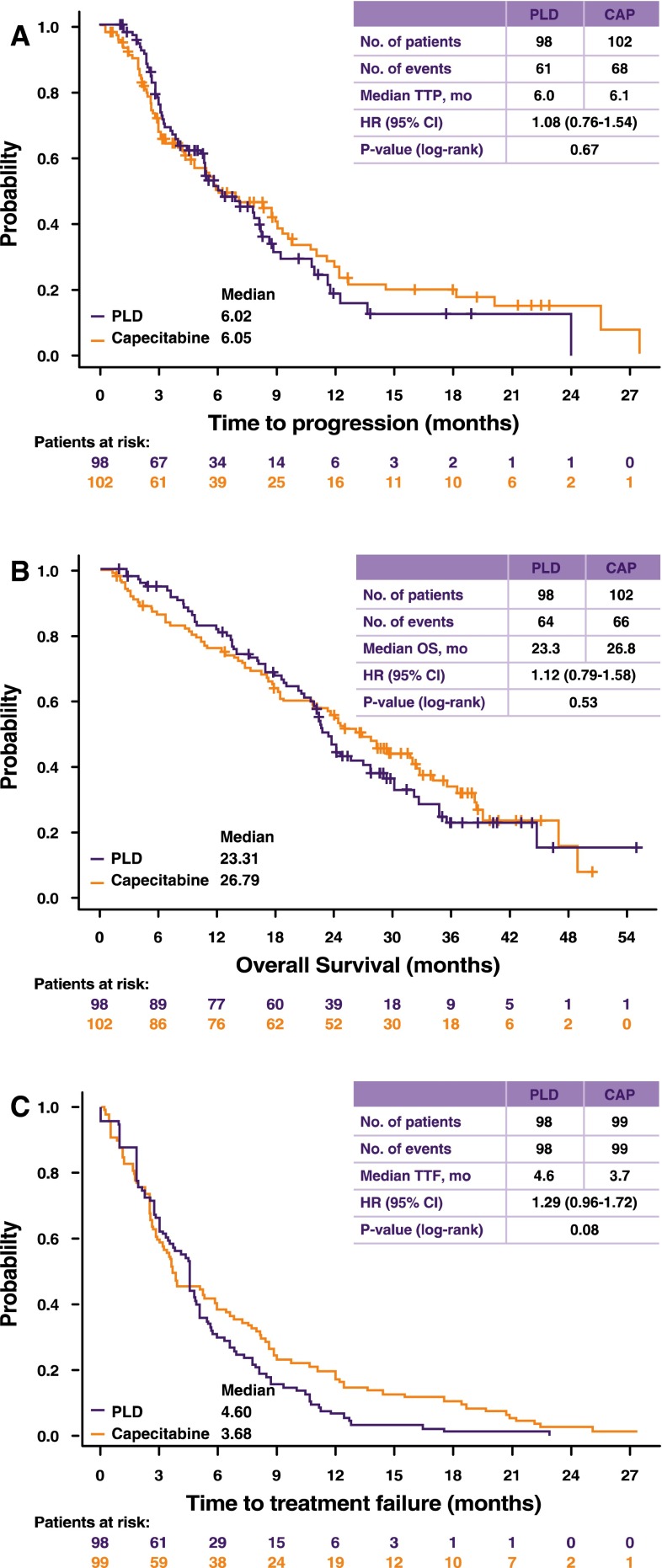
Time to disease progression (**a**), overall survival (**b**), and time to treatment failure (**c**)

The primary endpoint, TTP, was not statistically significantly different between arms (Fig. 2a). The HR for PLD versus capecitabine was 1.08 (95 % confidence interval 0.76—1.54; *P* = 0.67). The upper bound of the one-sided 97.5 % CI for the HR of PLD is 1.54. Since this bound is greater than the bound for non-inferiority (1.143), PLD cannot be considered non-inferior to capecitabine. When analyzed by prior adjuvant anthracycline administration (Table 2), TTP in the PLD arm remained similar. In the capecitabine arm, patients who had received prior anthracyclines had a statistically significantly shorter TTP compared to those without prior anthracyclines (median TTP 4.8 months vs. 8.3 months; log-rank *P* = 0.04).

**Table 2:** Time to progression by prior adjuvant anthracycline administration

	PLD *N* = 98		Capecitabine *N* = 102	
	Prior anthracycline			
	No	Yes	No	Yes
Number of patients	61	37	64	38
Number of events	34	27	38	30
Median TTP (months)	7.1	5.8	8.3	4.8
Log-rank *P* value	0.64		0.04	
Proportion without progression (%)				
At 6 months	54	47	56	42
At 12 months	21	18	36	13

Both confirmed and unconfirmed overall response rates were similar between arms. The confirmed overall response rate according to investigator assessment was 7.3 % in the PLD arm (*n* = 82) and 13.8 % in the capecitabine arm (*n* = 87; *P* = 0.17). One complete response was observed in the PLD arm. The corresponding overall response rates according to RECIST were 10.7 % among 84 assessable patients on the PLD arm and 12.9 % among 85 assessable patients on the capecitabine arm (*P* = 0.65).

At the time of the analysis, 70 patients were still alive: 34 patients on the PLD arm (34.7 %) and 36 patients on the capecitabine arm (35.3 %). Overall survival was not statistically significantly different between treatments (Fig. 2b). Time-to-treatment failure was also similar between arms (Fig. 2c).

### Safety

The most common adverse events of any grade included PPE, stomatitis, and fatigue in the PLD arm and PPE, fatigue, and diarrhea in the capecitabine arm (Table 3). Patients receiving PLD experienced a greater incidence of all-grade leukopenia (38 vs. 17 %; *P* = 0.002), stomatitis (40 vs. 17 %; *P* = 0.0007), ear, nose and throat abnormalities (43 vs. 17 %; *P* < 0.0001), alopecia (28 vs. 10 %; *P* = 0.002), and constipation (26 vs. 10 %; *P* = 0.005); patients receiving capecitabine had higher rates of diarrhea (43 vs. 16 %; *P* < 0.0001), and pulmonary embolism (6 vs. 0 %; *P* = 0.04). More patients in the capecitabine arm experienced thromboembolism of any type (17 vs. 2 %). Serious adverse events (SAE) of any type were more common in the capecitabine arm. A total of 59 SAEs occurred in the PLD arm compared to 112 in the capecitabine arm. Because the treatment duration of capecitabine was longer than PLD, the adverse event incidence density was calculated and revealed a statistically significant difference in favor of PLD for SAEs (0.023, PLD vs. 0.037, capecitabine; *P* = 0.003). The incidence of cardiac events was not statistically significantly different between arms, irrespective of prior anthracycline exposure (Table 3); however, among patients with prior anthracycline exposure, the proportion of cardiac events was somewhat elevated in the capecitabine arm (18 vs. 8 %; *P* = 0.31). One patient in the PLD arm experienced a grade 5 cardiac event consisting of cardiac decompensation. Among patients with both a baseline and at least one post-baseline LVEF measurement (*n* = 86, PLD and *n* = 90, capecitabine), an absolute decrease in LVEF of ≥10 % occurred in 6 patients (7 %) in the PLD arm and 8 patients (9 %) in the capecitabine arm (*P* = 0.64); a decrease of ≥20 to <50 % occurred in no patients in the PLD arm and in 2 patients (2 %) in the capecitabine arm (*P* = 0.16).

**Table 3:** Adverse events

Adverse Event	PLD *N* = 98		Capecitabine *N* = 102		*P* ^a^
	All grades No. (%)	Grade 3–4 No. (%)	All grades No. (%)	Grade 3–4 No. (%)	
Hematologic Toxicity					
Leukopenia	37 (38)	4 (4)	17 (17)	1 (1)	.002
Anemia	25 (26)	1 (1)	21 (20)	5 (5)	.10
Neutropenia	18 (18)	3 (3)	10 (10)	2 (2)	.19
Thrombocytopenia	6 (6)	1 (1)	6 (6)	1 (1)	1.0
Non-hematologic toxicity occurring in ≥20 % of patients in either arm					
Hand-foot syndrome	65 (66)	38 (39)	69 (67)	27 (26)	.08
Stomatitis	39 (40)	6 (6)	18 (17)	0	.0007
Fatigue	53 (54)	4 (4)	55 (54)	7 (7)	.71
Ear, nose, throat abnormality	42 (43)	6 (6)	17 (17)	0	<.0001
Nausea	41 (42)	0	42 (41)	2 (2)	.59
Alopecia	27 (28)	–	10 (10)	–	.002
Constipation	25 (26)	0	10 (10)	0	.005
Vomiting	18 (18)	0	30 (30)	2 (2)	.09
Peripheral sensory neuropathy	17 (17)	1 (1)	25 (24)	0	.19
Diarrhea	16 (16)	0	44 (43)	13 (13)	<.0001
Dyspnea	14 (14)	3 (3)	24 (24)	7 (7)	.23
Cardiac events					
Total events	9 (9)	1 (1)	13 (13)	0	.50
Without anthracycline pretreatment					
Total no. of patients	61		64		
Patients with cardiac events, no. (%)	6 (10)	1 (2)	6 (9)	0 (0)	1.0^b^
With anthracycline pretreatment					
Total no. of patients	37		38		
Patients with cardiac events, no. (%)	3 (8)	0	7 (18)	0	0.31^b^

^a^ Fisher’s exact test for grade 0 versus 1–2 versus 3–5

^b^ Fisher’s exact test

### Quality of life

The two therapies generally had a similar effect on QoL. Mean changes from baseline in EORTC QLQ C-30 scores were significantly different between PLD and capecitabine at selected timepoints in certain domains (Table 4). For all SSQ domains, the majority of responses across all cycles were categorized as same or better since the last visit in both arms (Table 5).

**Table 4:** Mean Change from baseline in EORTC QLQ-C30

Domain	Parameter	Cycle 1		Cycle 2		Cycle 3		Cycle 4		EOT/EOS	
		PLD	CAPE	PLD	CAPE	PLD	CAPE	PLD	CAPE	PLD	CAPE
Physical functioning	No. patients	*n* = 19	*n* = 10	*n* = 52	*n* = 36	*n* = 48	*n* = 28	*n* = 30	*n* = 23	*n* = 5	*n* = 5
	Score change^a^	0	0	−1.0	−13.0	−2.8	−10.7	−0.7	−15.9	−10.7	−24.0
	*P* ^c^	.86		.01		.06		.006		.31	
Role functioning	No. patients	*n* = 19	*n* = 10	*n* = 51	*n* = 36	*n* = 48	*n* = 28	*n* = 30	*n* = 24	*n* = 5	*n* = 5
	Score change^a^	2.6	−3.3	−5.2	−15.3	−6.6	−14.3	−4.4	−22.2	−16.7	−23.3
	*P* ^c^	.33		.24		.25		.02		.67	
Cognitive functioning	No. patients	*n* = 19	*n* = 10	*n* = 52	*n* = 36	*n* = 47	*n* = 28	*n* = 30	*n* = 26	*n* = 5	*n* = 5
	Score change^a^	4.4	−13.3	−1.0	−0.9	2.8	1.8	−0.6	2.6	0	−23.3
	*P* ^c^	.04		.73		.98		.26		.18	
Global health status	No. patients	*n* = 16	*n* = 9	*n* = 45	*n* = 31	*n* = 43	*n* = 21	*n* = 27	*n* = 20	*n* = 5	*n* = 4
	Score change^a^	8.3	−6.5	6.5	−4.0	4.4	−2.0	0	−16.2	−13.3	2.1
	*P* ^c^	.25		.29		.63		.04		.99	
Fatigue	No. patients	*n* = 19	*n* = 10	*n* = 52	*n* = 36	*n* = 48	*n* = 29	*n* = 30	*n* = 25	*n* = 5	*n* = 5
	Score change^b^	−3.2	2.2	4.6	11.4	3.7	8.4	1.8	19.1	11.1	24.4
	*P* ^c^	.41		.49		.29		.02		.54	
Constipation	No. patients	*n* = 19	*n* = 8	*n* = 52	*n* = 35	*n* = 47	*n* = 27	*n* = 30	*n* = 23	*n* = 5	*n* = 5
	Score change^b^	−3.5	0	10.9	−2.8	10.6	−4.9	17.8	−13.0	0	−6.7
	*P* ^c^	.44		.07		.22		.004		.48	
Dermatology/skin	No. patients	*n* = 15	*n* = 9	*n* = 42	*n* = 28	*n* = 40	*n* = 20	*n* = 22	*n* = 19	*n* = 5	*n* = 3
	Score chang^b^	6.1	−5.6	9.1	1.2	33.3	12.1	37.1	22.8	18.3	44.4
	*P* ^c^	.08		.20		.04		.26		.70	

Includes only domains for which a statistically significant difference between arms was observed at any timepoint. Domains excluded are EORTC QLQ C30 Emotional Functioning, Social Functioning, Nausea/Vomiting, Pain, Dyspnea, Insomnia, Appetite Loss, Diarrhea and Financial Problems

*CAPE* capecitabine, *EORTC QLQ*-*C30* European Organisation for the Research and Treatment of Cancer Quality of Life Questionnaire C-30, *EOS* end of study, *EOT* end of treatment, *PLD* pegylated liposomal doxorubicin

^a^Mean change in score from screening; negative value indicates deterioration in QoL, positive value indicates improvement

^b^Mean change in score from screening; greater value indicates worse symptoms

^c^ANCOVA; for comparison between treatment arms in change from screening

**Table 5:** Proportion of responses to the subjective significance questionnaire (SSQ) across all cycles categorized as same or better since last visit

Domain	PLD	Capecitabine
No. responses across all cycles	522	708
Physical condition (%)		
Same	56.8	56.2
Better	14.8	19.8
Emotional condition (%)		
Same	58.8	57.3
Better	13.8	19.5
Social life enjoyment (%)		
Same	63.6	62.4
Better	13.2	16.5
Overall quality of life (%)		
Same	55.5	55.7
Better	14.3	18.2

Better category combines the following responses: “a little better”, “moderately better”, and “very much better”

*PLD* pegylated liposomal doxorubicin

## Discussion

The PELICAN trial evaluated for the first time the first-line efficacy of PLD and capecitabine in a randomized setting for all patients. A recent Dutch phase III trial compared first-line PLD versus capecitabine in elderly patients with MBC but had to be closed prematurely due to slow accrual and lack of supply of PLD. In the Dutch phase III trial, PLD and capecitabine demonstrated comparable efficacy, the number of geriatric conditions correlated with grade 3–4 toxicities, and frailty correlated with shorter survival [24, 25]. In our phase III PELICAN trial, efficacy of first-line PLD was neither superior nor non-inferior to capecitabine in patients with MBC, as reflected by TTP (HR = 1.08; *P* = 0.67) and OS (HR = 1.12; *P* = 0.53). Notably, alopecia was absent in a majority of patients in both arms, making both therapies very attractive for first-line use. Other acute toxicities differed between the two agents with more leucopenia and mucositis of any grade occurring with PLD and diarrhea and thromboembolic events of any grade and of high grades occurring more frequently with capecitabine. Patients in the capecitabine arm experienced a higher rate of serious adverse events and a non-statistically significant elevation in cardiac events among those with previous anthracycline exposure. One high-grade cardiac event was observed, occurring in the PLD arm.

The efficacy observed with PLD in this study was consistent with prior first-line experience. Median TTP of 6.0 months and median OS of 23.3 months were similar to those observed in the trial of PLD versus doxorubicin in which patients on PLD experienced a median PFS of 6.9 months and median OS of 21 months [6]. Conversely, the efficacy of capecitabine (median TTP, 6.1 months; median OS, 26.8 months) was somewhat more favorable compared to median TTP between 3.9 and 7.4 months in prior first-line studies [8, 22–26]. These differences may account for the lack of superiority of PLD over capecitabine in the current trial.

With the widespread use of adjuvant anthracyclines, repeat use of these agents in the metastatic setting has warranted caution due to potential reduction in efficacy and increased cardiotoxicity. The PELICAN trial addressed these key issues. Results clearly demonstrated that the benefit from PLD was present regardless of prior anthracycline use (median TTP 7.1 months with prior exposure versus 5.8 months without; *P* = 0.64). Surprisingly, prior anthracycline use affected the efficacy of capecitabine; those without prior anthracycline exposure had a statistically significantly higher TTP than those with previous exposure (*P* = 0.04; median 8.3 vs. 4.8 months). The difference between groups is driven by an unexpectedly long TTP in unexposed patients; in phase II trials that included fewer than 30 % of anthracycline-pretreated patients, median TTP with first-line capecitabine ranged from 3.9 to 6 months [8, 16, 22].

Cardiac safety data from the current trial corroborates the favorable profile of PLD, and allays fears of increased cardiotoxicity with use following prior anthracycline exposure. The trial demonstrated no increase in overall cardiac events in the PLD arm compared to the capecitabine arm. In fact, among patients with prior anthracycline exposure, a numerically higher rate of cardiac events occurred in the capecitabine arm (18 vs. 8 %; *P* = 0.31). The high-grade cardiac event in the PLD arm occurred in a patient without prior anthracycline treatment. The current trial required patients to enter without evidence of clinical CHF and a LVEF of ≥50 %. Thus, patients who had persistent cardiac decompensation following prior anthracycline use were excluded while anthracycline-naïve patients were not subject to a therapeutic trial that might select individuals particularly sensitive to cardiotoxicity. These results highlight the need for biomarkers to predict which patients are most susceptible to anthracycline-induced cardiac damage.

The primary endpoint (TTP) was similar between PLD and capecitabine (median TTP, 6.0 months versus 6.1 months, respectively). PLD failed to demonstrate superiority as first-line therapy in unselected patients with MBC. Use of a prior anthracycline did not affect the TTP of patients treated with PLD, a particularly relevant outcome as most patients today have received an anthracycline-taxane regimen in the adjuvant setting. The lack of alopecia and similar QoL observed with both agents make each agent a favorable first-line treatment option. It can be concluded from the PELICAN trial that both PLD and capecitabine are active and represent effective and relatively well-tolerated treatment options for first-line MBC. For the individual patient, chemotherapy choice may depend on the physician’s and patient’s preferences, with consideration given to prior adjuvant therapy as well as each drug’s safety profile.
